# Different phase delays of peripheral input to primate motor cortex and spinal cord promote cancellation at physiological tremor frequencies

**DOI:** 10.1152/jn.00935.2012

**Published:** 2014-02-26

**Authors:** Saša Koželj, Stuart N. Baker

**Affiliations:** Institute of Neuroscience, Newcastle University, Newcastle upon Tyne, United Kingdom

**Keywords:** coherence, motor cortex, oscillations, somatosensory input, spinal cord

## Abstract

Neurons in the spinal cord and motor cortex (M1) are partially phase-locked to cycles of physiological tremor, but with opposite phases. Convergence of spinal and cortical activity onto motoneurons may thus produce phase cancellation and a reduction in tremor amplitude. The mechanisms underlying this phase difference are unknown. We investigated coherence between spinal and M1 activity with sensory input. In two anesthetized monkeys, we electrically stimulated the medial, ulnar, deep radial, and superficial radial nerves; stimuli were timed as independent Poisson processes (rate 10 Hz). Single units were recorded from M1 (147 cells) or cervical spinal cord (61 cells). Ninety M1 cells were antidromically identified as pyramidal tract neurons (PTNs); M1 neurons were additionally classified according to M1 subdivision (rostral/caudal, M1r/c). Spike-stimulus coherence analysis revealed significant coupling over a broad range of frequencies, with the strongest coherence at <50 Hz. Delays implied by the slope of the coherence phase-frequency relationship were greater than the response onset latency, reflecting the importance of late response components for the transmission of oscillatory inputs. The spike-stimulus coherence phase over the 6–13 Hz physiological tremor band differed significantly between M1 and spinal cells (phase differences relative to the cord of 2.72 ± 0.29 and 1.72 ± 0.37 radians for PTNs from M1c and M1r, respectively). We conclude that different phases of the response to peripheral input could partially underlie antiphase M1 and spinal cord activity during motor behavior. The coordinated action of spinal and cortical feedback will act to reduce tremulous oscillations, possibly improving the overall stability and precision of motor control.

effective control of movement requires integration of sensory input to ensure successful performance of the task goal (Todorov and Jordan 2002). Reflecting this need for feedback, many “motor” areas of the nervous system show clear sensory receptive fields. Spontaneous neural oscillations may provide a useful model system to study interactions between sensory and motor activity. Within the primary motor cortex (M1) of primates endogenous activity occurs in two main frequency bands: the mu and beta rhythms, with frequencies at ∼10 Hz and ∼25 Hz, respectively. Beta-band oscillations are seen in field potential or scalp recordings from motor cortex and are coherent with similar oscillations in contralateral contracting muscles (Baker et al. 1997; Conway et al. 1995). Peripheral oscillations activate afferent feedback (Baker et al. 2006; Wessberg and Vallbo 1995), which is returned to the somatosensory cortex (Witham et al. 2007, 2010, 2011; Witham and Baker 2007). During epochs of beta-band oscillations, somatosensory evoked potentials are increased in amplitude, suggesting enhanced sensorimotor integration (Lalo et al. 2007). It is possible that a sensorimotor loop in the beta band plays a functional role in “recalibrating” the state of the periphery after a movement has occurred (Baker 2007; Witham and Baker 2012) or, alternatively, in promoting a stable motor output (Gilbertson et al. 2005; Kristeva et al. 2007). During active compensation for slowly varying forces, higher-frequency gamma-band (∼40 Hz) corticomuscular coherence appears (Omlor et al. 2007).

Beta and gamma frequencies above 20 Hz can usually be propagated to muscle activity with only minor consequence for overt output, because the twitch times of most muscles are slow (50–100 ms; Burke 1981) and limb mechanics are sluggish. This imposes a low-pass filtering and ensures that oscillations in the electrical activity of muscle do not usually translate into behaviorally significant tremor. By contrast, slower central oscillations at ∼10 Hz are responsible for the dominant component of physiological tremor, which can be a major limitation to the precision of motor output. We recently investigated the relation of neural activity to tremor around 10 Hz in various key motor centers (Williams et al. 2010) in monkeys performing a slow finger movement that generates especially high peripheral oscillations in this band. Activity in M1, reticular formation, and cerebellum showed similar phase relationships to the tremor, whereas spinal cord interneurons fired out of phase with the brain centers. We suggested that convergence of activity from descending pathways and spinal interneurons on motoneurons could lead to phase cancellation, and a reduction in tremor amplitude.

Spinal interneurons form a rich and diverse substrate for motor processing (Fetz et al. 2002; Jankowska 2008). Many interneurons receive inputs from different classes of peripheral sensory receptors (Bannatyne et al. 2009), as well as from descending axons such as the corticospinal and reticulospinal tract (Riddle and Baker 2010). An important unresolved question concerns the neural mechanisms that lead to antiphase activity in spinal interneurons compared with brain centers, since the rich connectivity admits many possible circuits. One clue comes from a detailed examination of the oscillatory coupling between peripheral tremor and motor cortical field potentials (Williams et al. 2009). The relative phases between the periphery and cortex suggest that the dominant process is sensory feedback from the periphery rather than descending outflow of motor commands. This conclusion is supported by directed coherence (Granger causality) analysis: while interactions around 10 Hz can be seen in both the local field potential (LFP) → tremor and tremor → LFP directions, the later dominate. If sensory feedback were configured subtly differently to spinal interneurons and motor cortical cells (e.g., with different delays or different proportions of suppression and facilitation), this could conceivably generate the observed phase difference during voluntary movement. Examination of the responses to sensory feedback is complicated if this occurs in the context of a sensorimotor loop, as during natural behavior. One possible solution is to measure responses to imposed peripheral inputs; because these are not determined by the animal's voluntary movement, this can allow measurements closer to the “open loop” condition (Schouten and Campfens 2012).

Previous work has reported the responses of motor cortical neurons to a variety of peripheral inputs (Cheney and Fetz 1984; Lemon and Porter 1976; Lucier et al. 1975; Rosen and Asanuma 1972). For spinal cord interneurons, the response to peripheral input is often used to identify different interneuronal classes. However, no previous reports have considered these sensory responses in the frequency domain, which is necessary to reveal possibly functionally important phase differences.

In the experiments reported here we stimulated peripheral nerves in the upper limb of macaque monkeys while recording neural spiking from the motor cortex or spinal cord. The stimuli were timed as a Poisson train, which has spectral power over a broad band; coherence analysis was used to identify coupling at different frequencies (Witham and Baker 2011). The animals were sedated or anesthetized, preventing voluntary movements. We find that there are indeed phase differences between sensory inputs to the spinal cord and identified corticospinal neurons in the motor cortex. Many M1 cells, especially those located in the bank of the central sulcus, had a phase difference relative to spinal cord cells close to π radians at ∼10 Hz. Sensory feedback may therefore be configured to promote phase cancellation at frequencies relevant to physiological tremor.

## METHODS

### Surgical Preparation

Experiments were carried out on two adult female *Macaca mulatta* monkeys (*monkey T*, weight 7.7 kg; *monkey Z*, weight 5.8 kg). All experiments were approved by the Newcastle University Animal Welfare and Ethical Review Board and performed under appropriate licenses from the UK Home Office. An initial implant surgery prepared the animals for subsequent chronic recording under general anesthesia (sevoflurane inhalation 2.7–4.0% and alfentanil 10 μg·kg^−1^·h^−1^ iv infusion). Methylprednisolone (loading dose 30 mg/kg, followed by 5.4 mg·kg^−1^·h^−1^ iv infusion) was administered to reduce brain edema. The nonsteroidal anti-inflammatory drug (NSAID) carprofen (4 mg/kg im) and prophylactic antibiotics (Clamoxyl LA, 0.1 mg/kg im) were administered prior to surgery onset. Animals were intubated to ensure a clear airway and artificially ventilated throughout surgery. Intravenous fluids (Hartmann's solution, 5 ml·kg^−1^·h^−1^) were administered to ensure fluid balance. Vital sign monitoring included pulse oximetry, heart rate, end-tidal CO_2_, noninvasive arterial blood pressure, and skin and core temperature. Temperature was maintained via a thermostatically controlled heating pad under the animal and a blanket supplied with air warmed to 38°C.

Under aseptic conditions, both animals were implanted on the right side with bipolar cuff electrodes around the median and ulnar nerves in the upper arm and the deep and superficial radial nerves at the elbow. Wires from these electrodes were routed subcutaneously to a connector placed on the head. *Monkey T* was fitted with an annular headpiece made from TecaPEEK and custom designed to fit the skull on the basis of a prior MRI scan. A stainless steel recording chamber was positioned over a craniotomy targeting the left motor cortex (target stereotaxic coordinates of chamber center A12 L18). Bolts attached to the headpiece allowed subsequent atraumatic head fixation. *Monkey Z* was implanted with two stainless steel chambers, located over the left and right motor cortex (coordinates as in *monkey T*). These chambers were attached to the skull with miniature stainless steel brackets, which were held to the bone with titanium skull screws (Synthese part no. 402.008; 5 screws per chamber). Subsequent head fixation of this animal used metal brackets that attached to holes within the chambers. In the same surgery, two small craniotomies were made just anterior to the chamber for subsequent implantation of pyramidal tract (PT) electrodes and their stereotaxic coordinates measured. These craniotomies were then covered with Gelfoam and sealed with a thin layer of dental acrylic. At the end of the implant surgery, animals were given a single dose of buprenorphine (5 μg/kg). Postoperative care included daily NSAID (meloxicam, oral suspension 90 μg/kg daily for 5 days) and antibiotic (Clamoxyl LA, 15 mg/kg im every 2 days for 2 wk) treatment.

In *monkey Z*, after conclusion of recordings from the left motor cortex (right arm stimulation), a further surgery implanted cuff electrodes in the left arm over the same four peripheral nerves as used on the right. Craniotomies were then opened in the chamber over right M1 and for right PT electrode implant, and further recordings were gathered.

### Motor Cortical Recordings

After recovery from the implant surgery was complete, regular experiments were carried out under light sedation. Animals were initially sedated with ketamine (5 mg/kg im) and medetomidine (0.1 mg im) and transferred to the laboratory. After placement of an intravenous line, sedation was continued by continuous intravenous infusion of a mixture of these agents. The infused solution contained 10 mg/ml ketamine and 20 μg/ml medetomidine; infusion rates ranged from 1.5 to 3 ml/h. Supplemental intravenous doses of ketamine (5 mg) or medetomidine (50 μg) were given as necessary. During recording sessions animals breathed 100% oxygen via a face mask and respiration was monitored via a nasal catheter linked to a capnograph. Heart rate and oxygen saturation were monitored via pulse oximetry; animals were kept warm with a thermostatic heating pad. The head was fixed atraumatically with the implanted devices; no painful stimuli were given during these recordings. The aim of this regime was to maintain animals in a stable state of light sedation, without spontaneous movements. To enhance neural activity, anesthetic depth was maintained deliberately light, such that corneal and withdrawal reflexes were often present. We thus refer to this regime as sedation rather than anesthesia.

In the first such session after the implant surgery, two fine Parylene-insulated stainless steel electrodes (tip impedance ∼100 kΩ; Microprobe MF501G) were inserted into the PT at the medulla through the craniotomies previously made for this purpose. Initial target stereotaxic coordinates were anterior 2.0 mm, lateral 0.7 mm, depth −6.0 mm and posterior 3.0 mm, lateral 0.7 mm, depth −10.0 mm relative to the interaural line. Electrodes were inserted with a double-angle technique (Soteropoulos and Baker 2006). Stimuli were delivered through the electrodes as they were advanced (300 μA, biphasic pulses, 0.1 ms per phase), and responses were monitored in a recording taken from the dural surface overlying M1. Each electrode was fixed at the location with lowest threshold to evoke an antidromic field potential (onset latency ∼0.7 ms). Thresholds ranged from 20 to 80 μA (mean 52 μA).

Subsequent recording sessions used an Eckhorn microdrive (Thomas Recording, Marlburg, Germany) to insert up to five glass-insulated platinum electrodes into M1. Electrodes were positioned to record spontaneously active cells that could be antidromically activated from the PT. The antidromic nature of the activation was confirmed by its fixed latency and by a collision test (Baker et al. 1999). After isolation of cleanly discriminable cells, stimuli were given through the four implanted peripheral nerve electrodes. For the median, ulnar, and deep radial nerves, an intensity just below motor threshold was used. For the superficial radial nerve, the stimulus intensity was two to three times the threshold for eliciting a field potential response in the cortex. Stimuli were timed as independent Poisson processes to the four nerves, with a mean rate for each nerve of 10 Hz. Neural waveforms were amplified (gain 2–10 K, band pass 300 Hz–10 kHz) and sampled continuously to computer hard disk at 25 kSamples/s, together with markers indicating the timing of stimuli. Recordings were typically gathered for 1,000 s.

On many days multiple sets of cells were recorded responding to the peripheral nerve stimulation. At the end of each session the antimitotic agent 5-fluorouracil was administered inside the chamber to reduce dural growth (Baker et al. 1999; Spinks et al. 2003) before the chamber was sealed. A single dose of atipamezole (0.5 mg im) was then given to reverse the medetomidine. Recovery was rapid and uneventful; animals were usually eating within 10 min and able to return to their cage mates within an hour. Recording sessions typically lasted 4–6 h and were carried out three times per week. Such regular sedation sessions seemed to have minimal impact on the monkeys' welfare, and animals maintained their weight during the recording period. We attribute this to the low drug doses required when using continuous intravenous infusion, the careful attention paid to maintaining the animal's temperature and blood oxygenation throughout, and the ability for rapid pharmacological reversal of sedation at the end of the session.

### Spinal Cord Recordings

After data acquisition from M1 was complete, recordings were made from the spinal cord in a surgical procedure under terminal general anesthesia. Initial anesthesia followed the regime described above for the implant surgery. The animal was additionally prepared with a tracheotomy, and arterial and venous catheters were inserted into the major neck vessels on one side for continual monitoring of blood pressure. A laminectomy was performed to expose spinal segments C_7_ to T_1_. The spinal column was clamped in a spinal frame at the high thoracic and lumbar levels, and the head was fixed in a stereotaxic frame angled to provide ∼60° of neck flexion. The spinal dura mater was removed. Anesthesia was then switched to an intravenous infusion of propofol (25–50 mg·kg^−1^·h^−1^) and alfentanil (10–70 μg·kg^−1^·h^−1^). Neuromuscular blockade was initiated by administering atracurium (initial dose 0.8 mg/kg, followed by infusion of 0.7 mg·kg^−1^·h^−1^). Throughout the procedure heart rate and arterial blood pressure were continually monitored. Slow increasing trends, or more rapid rises in response to noxious stimuli, were taken as evidence of lightening anesthetic depth. Supplementary doses of anesthetic were then given and infused rates increased accordingly.

An Eckhorn microdrive was used to introduce up to 16 microelectrodes into the spinal cord. Once cleanly discriminable and spontaneously active single-unit activity was isolated on multiple channels, recordings were made of the responses to stimulation through the ipsilateral nerve cuff electrodes (intensities just below motor threshold for median, ulnar, or deep radial nerves, 3 × threshold to elicit a spinal volley for superficial radial nerve) and the contralateral PT electrodes (300 μA, biphasic pulses, 0.1 ms per phase). All five stimuli were delivered as independent Poisson trains, mean rate 10 Hz.

At the end of the spinal recordings the anesthetic depth was increased by an overdose of anesthetic, and the animal was perfused through the heart with phosphate-buffered saline, followed by formalin. Spinal and cortical tissue was then removed and placed in ascending concentrations of sucrose solution for cryoprotection (final concentration 30%). Tissue was cut on a freezing microtome (50-μm sections) and stained with cresyl violet. Representative sections were photographed and traced to allow reconstruction of cortical and spinal recording sites based on the microdrive coordinates noted during the recording sessions.

### Data Analysis

#### Spike detection and correction for stimulus artifacts.

**Fig. 1.**
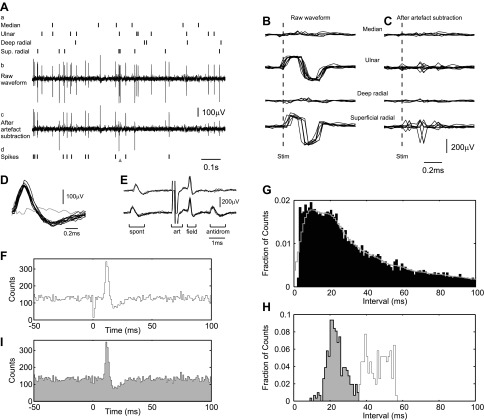
Example data, illustrating analysis stages. *A*: 1 s-long section of recording, showing stimulus times of 4 peripheral nerve electrodes (*a*), recording from an electrode placed within primary motor cortex (M1) (*b*), waveform of *b* after processing to subtract average stimulus artifacts (*c*), and extracted spike times (ticks) and the time at which a spike was inserted to reduce artifactual reduction of rate during stimulus artifact dead line (arrowhead) (*d*). *B*: overlain stimulus artifacts corresponding to the different nerve stimuli shown in *A*. *C*: residual artifacts after the mean artifact has been subtracted. *D*: overlain spike waveforms corresponding to times marked in *Ad*. Gray trace corresponds to the time of the arrowhead in *Ad*. *E*: collision test. Pyramidal tract stimulation (art) was triggered at a fixed delay after a spontaneous spike (spont). Stimulus led to a field potential response (field) and an antidromic spike (antidrom). The antidromic response was collided when the interval between spontaneous spike and stimulus was reduced to less than a critical value (*top*). *F*: peristimulus time histogram (PSTH) of spike shown in *D*, compiled relative to ulnar nerve stimulation. Note artifactual dip around *time zero*. *G*: raw interspike interval histogram (black bars) and smoothed curve (gray line; Gaussian kernel smooth, width parameter 3 ms). *H*: distribution of selected interspike intervals before (white) and after (gray) addition of a spike at the time of a stimulus. *I*: PSTH as in *H* but compiled from modified spike train.

The first stage of any analysis of neural spike data is to extract the time of action potential occurrence from the raw waveform recordings. This is typically performed with cluster cutting approaches (Lewicki 1998). However, in the present data the high rate of stimulus delivery, coupled with large stimulus artifacts on some channels, made this initial stage challenging. Figure 1*Ab* illustrates a 1-s-long recording from M1. The times of peripheral stimuli are indicated in Fig. 1*Aa* by ticks. For ulnar and superficial radial nerve stimulation the stimulus artifact was large and comparable in size to the neural spikes. Figure 1*B* shows the artifacts on an expanded timescale.

To reduce the impact of these artifacts, we first generated an average of the waveform relative to each stimulus marker and then subtracted this average from the waveform at every time where that stimulus occurred. Figure 1*Ac* shows this modified waveform, corresponding to the same time period illustrated in Fig. 1*Ab*; overlain sections on an expanded timescale are shown in Fig. 1*C*, corresponding to Fig. 1*B*. It is clear that there is some improvement. However, jitter of the stimulus timing relative to the waveform sampling clock led to some misalignment between sweeps (especially apparent for the superficial radial nerve in this example). Additionally, during the peaks of the artifacts the neural amplifier saturated. Even though the artifact for the median nerve has been reduced to a small size by subtraction, any spike that occurred within this period would not be detected.

Figure 1*D* shows overlain waveforms for spikes discriminated from this processed signal (black lines). This cell was identified antidromically as a pyramidal tract neuron (PTN) (collision test illustrated in Fig. 1*E*). Figure 1*F* presents a peristimulus time histogram (PSTH) of the spiking compiled relative to the ulnar nerve stimulation. There was a clear facilitation of discharge with an onset latency (8.75 ms) appropriate for known conduction delays, followed by a suppression. However, at *time zero* there was also an apparent suppression; this was caused by the failure to detect spikes during the period when the stimulus artifact saturated the recording amplifier. In other recordings, an artifactual peak sometimes occurred close to *time zero*, reflecting erroneous classification of the residual artifact as a neural spike. It was important to correct for either of these errors, as they represent a correlation between spike and stimulus timing that would bias subsequent estimates of coherence phase.

This correction was carried out as follows. First, we measured the baseline spike rate over the period 20 ms to 1 ms before the stimulus. Note that since nerve stimuli were being given with random timing relative to the nerve chosen as the trigger, this “baseline” rate also included a proportion of spike generated in response to the stimuli not used as the trigger. We denoted the period from 0.2 ms before to 1.0 ms after the stimulus as the “artifact window”; this time period is so brief that for any given stimulus either zero or one spike will fall within it. The spike counts within the artifact window *C* were compared with those expected over this time given the baseline *B*, and the statistical significance of any deviation was assessed from the Poisson counting distribution. If there was no significant difference in counts (*P* > 0.0005), no correction was deemed necessary. If a difference was detected, our algorithm selected all the stimuli where a spike had occurred (for instances with an artifactual facilitation, *C* > *B*) or not occurred (for an artifactual suppression, *C* < *B*) within the artifact window. Assume that there were *N* such stimuli, and denote the time from the stimulus to the first preceding spike on trial *i* as *b*_*i*_ and the time from the stimulus to the first subsequent spike on that trial as *a*_*i*_. Furthermore, we compiled the interspike interval histogram from all detected neural spikes and normalized it to represent the probability of a given interval (Fig. 1*G*, black bars). This histogram was then smoothed by convolution with a Gaussian kernel (width parameter 3 ms) to yield an estimated probability density function *P*(*I*) for interspike interval *I* (Fig. 1*G*, gray line).

For situations with an artifactual facilitation (*C* > *B*), we needed to remove the spike from the artifact window in *C* − *B* out of the *N* sweeps where it occurred; this would then restore the counts to the *B* counts expected from the baseline. We chose the sweeps in which to do this by maximizing the improvement of the likelihood of the resulting interspike intervals. With the spike left within the artifact window on sweep *i*, there are two interspike intervals *a*_*i*_ and *b*_*i*_; the data likelihood is hence *P*(*a*_*i*_)*P*(*b*_*i*_). If we remove this spike, there is only one interspike interval *a*_*i*_ + *b*_*i*_, and the data likelihood is *P*(*a*_*i*_ + *b*_*i*_). The likelihood ratio *R*_*i*_ specifies how much more likely the edited data are compared with the raw spike train, where

(1) Ri=P(ai+bi)P(ai)P(bi)

We therefore chose the (*B* − *C*) sweeps with the largest *R*_*i*_ and removed the spike within the artifact window on these sweeps.

For situations with an artifactual suppression (*C* < *B*), we need to add in a spike within the artifact window in *B* − *C* of the *N* sweeps where there is no spike to restore the counts to the expected value *B*. In this case, sweep *i* of the unedited spike train has a single interval *a*_*i*_ + *b*_*i*_, and the data likelihood is *P*(*a*_*i*_ + *b*_*i*_). If we add in a spike on that sweep, the two intervals *a*_*i*_ and *b*_*i*_ will result; the data likelihood then becomes *P*(*a*_*i*_)*P*(*b*_*i*_). The likelihood ratio is then *R*_*i*_, where

(2) Ri=P(ai)P(bi)P(ai+bi)

We chose the (*B* − *C*) sweeps with largest *R*_*i*_ and inserted a spike within the artifact window on those sweeps; the exact timing of the spike within the artifact window was chosen randomly from a uniform distribution. For the illustrated example spike train, Fig. 1*H* shows the distribution of the intervals *a*_*i*_ + *b*_*i*_ for the sweeps where a spike was added (white bars), as well as the distribution of the intervals *a*_*i*_ and *b*_*i*_ for those sweeps (gray bars). These intervals fall closer to the peak of the interspike interval distribution (Fig. 1*G*), making these sweeps more likely with the addition of a spike in the artifact window. Figure 1*I* shows the PSTH compiled with the edited spike train; as expected, the artifactual suppression around *time zero* has been removed.

Note that the function of this spike train editing was to remove an artifactual association between the spike and stimulus timing on average. We do not claim that a spike necessarily did occur on every sweep in which it was inserted—rather that a similar number of spikes probably occurred, and that we have added them into the most likely sweeps. Figure 1*Ad* shows the edited spike train, which has an additional spike (arrowhead) compared with the original spike train (black). Examination of the waveform associated with this event does not show any evidence that a spike actually did occur at this time (Fig. 1*D*, gray trace). In this regard, our algorithm differs slightly from previous applications of interval-based spike train editing (Stein and Weber 2004).

**Fig. 2.**
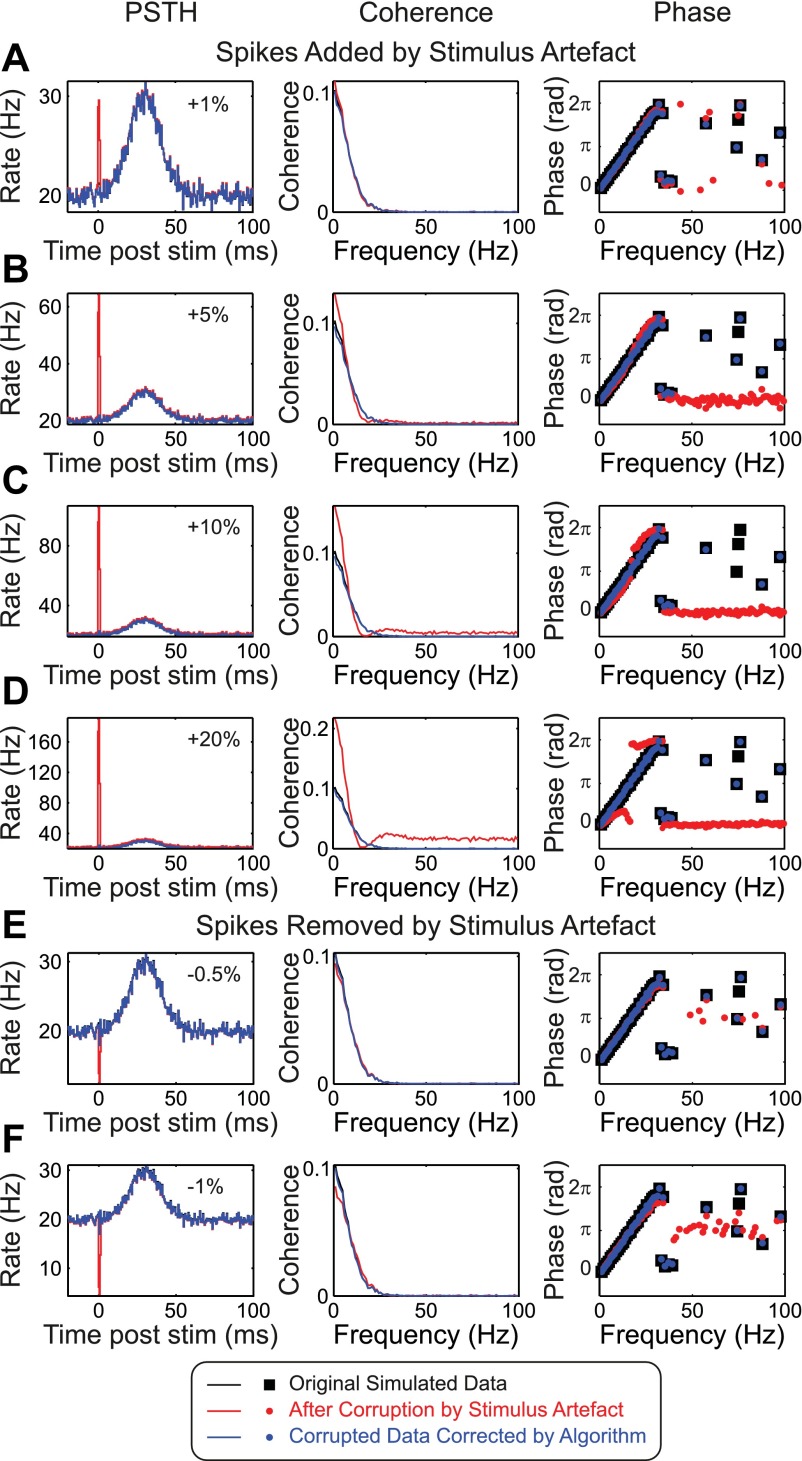
Effect of spike train contamination by stimulus artifact and of correction algorithm. Each row shows PSTH, coherence, and coherence phase plots generated from data simulated as described in the text. Black traces show results from original simulated data, before contamination by stimulus artifact; red traces show results after artifact contamination; blue traces show results from contaminated data after application of the correction algorithm described in the text and illustrated in Fig. 1. *A–D*: contamination took the form of addition of a spike at the time of the stimulus to 1%, 5%, 10%, and 20%, respectively, of sweeps, chosen at random. *E* and *F*: contamination took the form of subtraction of a spike at the time of the stimulus in 0.5% or 1%, respectively, of sweeps, chosen at random. Note the artifactual peak (*A–D*) or trough (*E* and *F*) in the PSTH around *time zero* seen in the red trace, reflecting the spike train contamination. In most cases, blue and black traces overlie so completely that the black trace cannot be seen.

Figure 2 illustrates the effect that this algorithm had on measures of coherence and phase, using simulated data. To compile this figure, we first generated stimulus event times as a Poisson process (mean rate 10 Hz). A continuous curve representing spike rate was then generated. This had a baseline rate of 20 Hz; whenever a stimulus event occurred, the spike rate transiently increased. The rate increase was shaped as a Gaussian curve, with a 30-ms delay from stimulus time to peak rate, a width parameter of 10 ms, and a maximal increase in rate of 10 Hz. The instantaneous rate profile was converted to a spike train by generating an inhomogeneous gamma process (order parameter 4) with the decimation procedure described previously (Baker and Gerstein 2000; Baker and Lemon 2000). This “uncorrupted” spike train formed the basis for estimating the effect of artifactual contamination of the data, and the success of our correction algorithm.

Figure 2, *A–D*, illustrate the effects of adding additional spikes in the vicinity of the stimulus artifact; the different panels show situations in which 1%, 5%, 10%, and 20% of stimuli generated artifactual extra spikes. Each panel presents the PSTH (*left*), coherence (*center*), and coherence phase (*right*) (see below for details of calculation), with overlain curves indicating the results from the original uncorrupted data (black), the spike train with artifactual extra spikes (red), and the spike train corrected by the algorithm described above (blue). As expected, the PSTH showed an artifactual peak around *time zero* in the red traces, which was successfully removed by the algorithm. The coherence was increased by the artifactual spikes and also extended to higher frequencies; this was especially evident for the stronger contamination (Fig. 2*D*). The coherence phase also deviated from the uncorrupted values as the level of contamination increased, with the slope of the phase-frequency relationship becoming close to zero for the greatest contamination examined. Additionally, the increased coherence at high frequencies led to more coherence values rising above significance. Because phase is only valid when its associated coherence is significantly nonzero, this produced more estimates for phase at higher frequencies; these all tended to cluster around zero phase. The correction algorithm successfully recovered coherence and phase spectra closely matching the uncorrupted data (compare blue and black traces in Fig. 2, *A–D*, which often overlie).

Figure 2, *E* and *F*, illustrate the converse condition, where we simulated removal of spikes caused by a failure to detect them during the stimulus artifact. In percentage terms, the maximum possible contamination of the spike train was smaller than in the additive case, because of a floor effect: when 1% of stimuli led to deletion of near-coincident spikes, this led to almost no spikes present around the stimulus time (Fig. 2*F*). Coherence and phase spectra were noticeably altered by the contamination, with extra phase estimates at high frequencies now tending to cluster around π radians. As in Fig. 2, *A–D*, application of the algorithm described above restored PSTH, coherence, and phase measures close to those made from the uncorrupted spike train.

The simulations in Fig. 2 used a baseline neural firing rate of 20 Hz. We repeated the analysis using rates of 5 Hz and 100 Hz (not illustrated), with similar results. We conclude that coherence measures made from spike trains where the stimulus artifact causes discrimination errors could be biased but that this can be corrected with the approach described in this section.

#### Time-domain analysis.

PSTHs of spike activity relative to each stimulus were compiled with a 0.5-ms bin width (Fig. 1, *F* and *I*). The Poisson processes used to drive stimulation of each nerve were independent. This meant that if the PSTH was triggered from stimuli given to one nerve, responses to activation of the other nerves would occur at random relative to the trigger point. Such responses would thus merely increase the apparent background activity level in the PSTH rather than generating any response locked to the triggering stimulus. Histograms were displayed to the experimenter, who placed interactive cursors to indicate the onset and offset of a possible response region. Many PSTHs showed multiple phases of response, for example, facilitation followed by suppression; only the first response was measured in these cases. These cursors were positioned guided by lines that indicated the baseline, and baseline ± 2SD, as well as the CUSUM (Ellaway 1978). The statistical significance of this region was assessed with the test given by Cope et al. (1987). We computed

(3) $$Z=\left(\frac{C_{P}}{N_{P}}-\frac{C_{B}}{N_{B}}\right)/\sqrt{\left(\frac{C_{P}}{{N_{P}}^{2}}-\frac{C_{B}}{{N_{B}}^{2}}\right)}$$

where the response and baseline regions span *N*_P_ and *N*_B_ bins, respectively, and contain *C*_P_ and *C*_B_ counts. Assuming that the bin counts follow a Poisson distribution, *Z* will be approximately normally distributed with mean 0, standard deviation 1 on the null hypothesis that spiking rate within the response is the same as in the baseline. A response was considered significant if |*Z*| > 3.29, corresponding to *P* < 0.001; this conservative criterion was used to correct for the multiple comparisons implied by the experimenter choosing the most plausible region to test.

#### Frequency-domain analysis.

To estimate the coherence between a stimulus and spike train, we followed methods used in our previous work (Witham and Baker 2011). Each pulse train was first converted to a waveform sampled at 1 kHz by counting events in 1-ms bins; these continuous recordings were segmented into 1,024-point-long nonoverlapping windows. If the Fourier transform of the stimulus on the *i*th window is denoted *X*_*i*_(*f*) and of the spike train *Y*_*i*_(*f*), the coherence is estimated as

(4) $$\text{Coh}(f)=\frac{{\left|\sum_{i=1}^{L}X_{i}(f)Y_{i}^{*}(f)\right|}^{2}}{\sum_{i=1}^{L}X_{i}(f)X_{i}^{*}(f)\sum_{i=1}^{L}Y_{i}(f)Y_{i}^{*}(f)}$$

where *L* is the number of data sections available. Coherence was assumed significantly different from zero (*P* < 0.05) if it was larger than *C*_lim_, where

(5) $$C_{\lim}=1-0.05^{1/(L-1)}$$

Results were combined across cells by averaging the coherence spectra; significance limits for the averaged coherence were determined as described by Evans and Baker (2003).

The coherence phase θ(*f*) was determined from the cross spectrum:

(6) $$\theta (f)=\arg\left(\sum_{i=1}^{L}X_{i}(f)Y_{i}^{*}(f)\right)$$

where arg (·) denotes the phase of the complex number representing the cross spectrum.

Phase spectra were subjected to linear regression analysis to test for linear phase-frequency relationships, which would be compatible with a fixed delay. In cases in which a significant linear relationship was found, the corresponding delay in seconds was calculated as

(7) $$\text{Delay}=\frac{b}{2\pi }$$

where *b* is the slope of the best fit line (in radians/Hz).

Phase was combined across a population of cells in two ways. The circular average of phase was calculated for each frequency bin using only cells *j* where coherence in that bin was significantly different from zero:

(8) $$\overset{¯}{\theta }(f)=\arg\left(\sum_{j\in {\text{Coh}}^{j}(f)>C_{\lim}^{j}}e^{i\theta^{j}(f)}\right)$$

Second, in order to represent possible heterogeneity of phase across the cell population, we defined the frequency band of interest for physiological tremor as 6–13 Hz (Williams et al. 2009, 2010). Within this band, if any cell had coherence significantly different from zero in a frequency bin, the phase of that bin was added into a list. The list of phases so compiled included multiple contributions from each cell, depending on how many coherence values rose above significance. The distribution of phases was then displayed as a circular histogram.

Phases were compared between cell classes with a shuffling approach. Unlike available analytical statistical methods, this has the advantage of being applicable regardless of the number of cells that contributed a phase to a given average phase estimate (Fisher 1993). The absolute difference between the circular-averaged phases of the two classes was computed. The individual values used to compute the averages were then shuffled between the classes and the absolute difference in average phase recomputed. This procedure was repeated 1,000 times; if the phase difference found from the original data exceeded 950/1,000 of the differences found after shuffling, the phases were assumed significantly different (*P* < 0.05).

## RESULTS

### Available Recordings

**Fig. 3.**
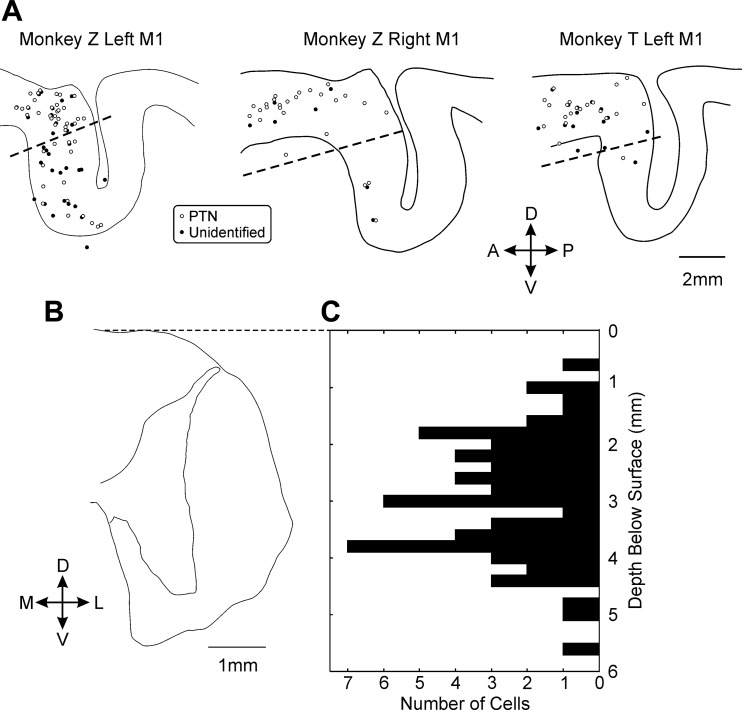
Anatomical location of recorded neurons. *A*: location of recorded cells in M1, superimposed on traced representative histological sections. Dashed line indicates the boundary used to separate M1r and M1c. PTN, pyramidal tract neuron. *B*: traced histological section of cervical spinal cord. *C*: histogram showing the distribution of the depth of recorded spinal cells below the cord surface, aligned to the tracing of *B*. Scales of histological sections have not been adjusted to account for possible minor tissue shrinkage following fixation.

Cleanly discriminable activity was available from 11, 14, and 13 multiple-electrode penetrations in *monkey T* left M1, *monkey Z* left M1, and *monkey Z* right M1, respectively; a total of 147 cells were discriminated, of which 90 were antidromically identified PTNs. Figure 3*A* shows the reconstructed location of the recording sites, superimposed on tracings of representative histological sections of the central sulcus in each case. The line on these plots indicates the boundary used to divide cells recorded in the bank of the sulcus (M1c) and the surface (M1r); this was placed with reference to figures from Rathelot and Strick (2006). The majority of available recordings from M1c came from *monkey Z* left M1.

Cleanly discriminable activity was recorded in six multiple-electrode penetrations into the cervical cord from each monkey, yielding a total of 61 cells. We found that small errors in estimation of the lateral location of the electrode tip during the experiment made a two-dimensional reconstruction of electrode locations overlain on a representative histological section unreliable. Accordingly, Fig. 3*C* shows only a histogram of the depth of recorded cells, at the same scale as a histological tracing (Fig. 3*B*). It is clear that recordings spanned the different spinal laminae but were mainly concentrated in the intermediate zone where interneurons are located. These recordings were made from anesthetized animals, at an anesthetic depth where there was no muscle tone prior to the induction of neuromuscular block. We would therefore not expect motoneurons to be tonically active, and hence they are not likely to be represented in our data set.

Of the entire available database, 93/208 spike trains (45%) were found to be corrupted by stimulus artifact contamination; 84 units had an artifactual reduction in rate around the time of the stimulus (as in Fig. 2, *E* and *F*), and 9 had an artifactual increase (Fig. 2, *A–D*). The correction algorithm described in methods led to addition or subtraction of an average of 1.4% of the total spikes (range 0.30–3.47%).

### Peristimulus Time Histograms

**Fig. 4.**
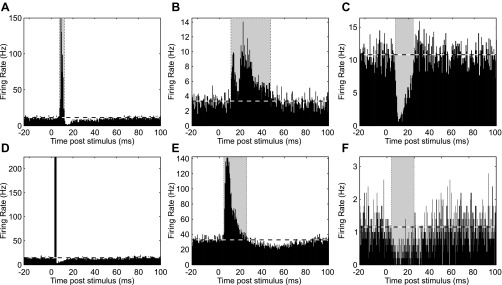
Example PSTHs. *A–C*: M1 cells (*A*, PTN from M1r, antidromic latency 0.9 ms; *B*, unidentified cell from M1c; *C*, PTN from M1c, antidromic latency 1.0 ms). *D–F*: spinal cord cells. Triggering stimuli: superficial radial nerve (*B*) or median nerve (*A*, *C–F*).

Past attempts to characterize cell responses to somatosensory input have used time-domain analysis, which reveals the range of responses (both facilitation and suppression) present at different latencies. Accordingly, we began by examining our data in this way. Figure 4 illustrates PSTHs compiled for example cells from M1 (Fig. 4, *A–C*) and spinal cord (Fig. 4, *D–F*). Some cells responded to the peripheral stimulation with a powerful, short-latency and brief facilitation of their discharge (Fig. 4, *A* and *D*). Such PSTH peaks were usually followed by a period of suppression; this may reflect a resetting of the cells' discharge and subsequent relative refractory period or a more active process of inhibition. A variety of response types was seen, including long-lasting facilitations (Fig. 4, *B* and *E*) that sometimes appeared to have multiple components (Fig. 4*B*). In some cases cells responded to the stimulation with a pure suppression of firing (Fig. 4, *C* and *F*).

**Fig. 5.**
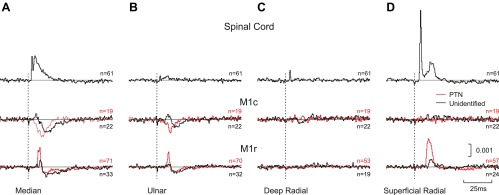
Population-averaged PSTHs. PSTHs were first normalized to provide the probability of a spike occurring in each 0.5-ms bin per stimulus, with the baseline (prestimulus) level of spiking subtracted. These normalized PSTHs were then averaged. Thin horizontal lines indicate the baseline level. Each row corresponds to a different neural center. Red lines indicate responses of antidromically identified PTNs and black lines unidentified cells. Vertical dashed line marks time of stimulus. *A*: median nerve. *B*: ulnar nerve. *C*: deep radial nerve. *D*: superficial radial nerve. *n*, Numbers of cells contributing to each average.

Figure 5 presents PSTHs averaged across the entire population of cells recorded from a given region. Although this masks important differences in responses between cells, it does provide a representation of how cell firing was affected on average. For the two flexor nerves (Fig. 5, *A* and *B*), it is striking that while spinal cord cells were facilitated on average, responses in M1c were dominated by suppression. For the unidentified cells, there appeared to be a small initial facilitation, followed by suppression; for the PTNs, only suppression could be seen in this population average. All M1r population averages showed a brief facilitation followed by a suppression after median or ulnar nerve stimulation. Responses to the stimulation of the deep radial nerve, which innervates forearm extensor muscles, were weak, and the population-averaged PSTHs showed only a small deviation for the spinal cord neurons (Fig. 5*C*). By contrast, stimulation of the cutaneous afferents of the superficial radial nerve (Fig. 5*D*) produced an average facilitation of spinal cord and M1r cells (which was stronger for PTNs than unidentified cells) but no consistent modulation of M1c cells.

Baseline firing rates, assessed from the prestimulus period in the PSTHs, were as follows: spinal cord, 11.8 ± 10.8 Hz; M1c PTNs, 8.6 ± 6.1 Hz; M1c unidentified, 9.1 ± 16.4 Hz; M1r PTNs, 8.2 ± 5.7 Hz; M1r unidentified, 8.3 ± 6.2 Hz (all means ± SD).

### Coherence Between Peripheral Stimulation and Neural Spiking

**Fig. 6.**
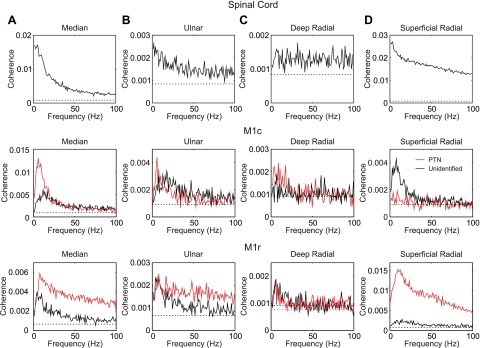
Population-averaged coherence spectra. Each trace illustrates the average coherence for a different neural population, in response to a specific peripheral nerve. Red traces show results for antidromically identified PTNs and black traces unidentified cells. Horizontal dashed lines indicate significance limits on the averaged coherence (*P* < 0.05). *A*: median nerve. *B*: ulnar nerve. *C*: deep radial nerve. *D*: superficial radial nerve. Numbers of cells contributing to each average spectrum are the same as given for the corresponding PSTH in Fig. 5.

To examine how cells represented stimulus information in the frequency domain, we used coherence analysis. Figure 6 presents coherence spectra between cell spiking and peripheral stimulation; spectra have been averaged over all cells of a given type recorded within the region. Coherence between spinal cord cells and median nerve stimuli (Fig. 6*A*) declined with frequency up to ∼50 Hz. Above this frequency, the average coherence remained above significance but was low. For M1c PTNs, the coherence peaked at 6.4 Hz, being greater than at lower or higher frequencies. It too then fell with increasing frequency, so that above 50 Hz it was close to the significance level. M1r PTNs showed lower levels of coherence, although there was also a peak at 8.3 Hz. Unidentified cells in both subdivisions of M1 showed a profile similar to that PTNs, although with a lower peak average coherence.

For the ulnar nerve (Fig. 6*B*) coherence levels appeared smaller, but findings were broadly similar as for the median nerve. Coherence with the deep radial nerve stimuli was very weak (Fig. 6*C*) and remained close to the significance limit at all frequencies; this agrees with the analysis using PSTHs, where little average response was also seen following this nerve (Fig. 5*C*). By contrast, cells from the spinal cord and M1r exhibited clear coherence with superficial radial nerve stimuli (Fig. 6*D*). In the case of M1r, average coherence was largest for PTNs, where it peaked at 10.3 Hz, whereas for the spinal cord it declined steadily with frequency. Unlike stimulation of the mixed nerves, coherence remained clearly above the significance limits even up to 100 Hz. Coherence between M1c PTNs and the superficial radial nerve was close to the significance limit at all frequencies. It was somewhat larger for unidentified cells but still very small compared with M1r PTNs or spinal cells. Once again, this agreed with the results from the time-domain analysis, as the averaged PSTH for M1c PTNs responding to this nerve showed no features (Fig. 5*D*).

The results of coherence analysis showed that cells in both spinal cord and M1 were capable of representing oscillatory information in peripheral input, especially at frequencies lower than 50 Hz.

### Comparison of Delay Estimates from Time- and Frequency-Domain Analyses

**Fig. 7.**
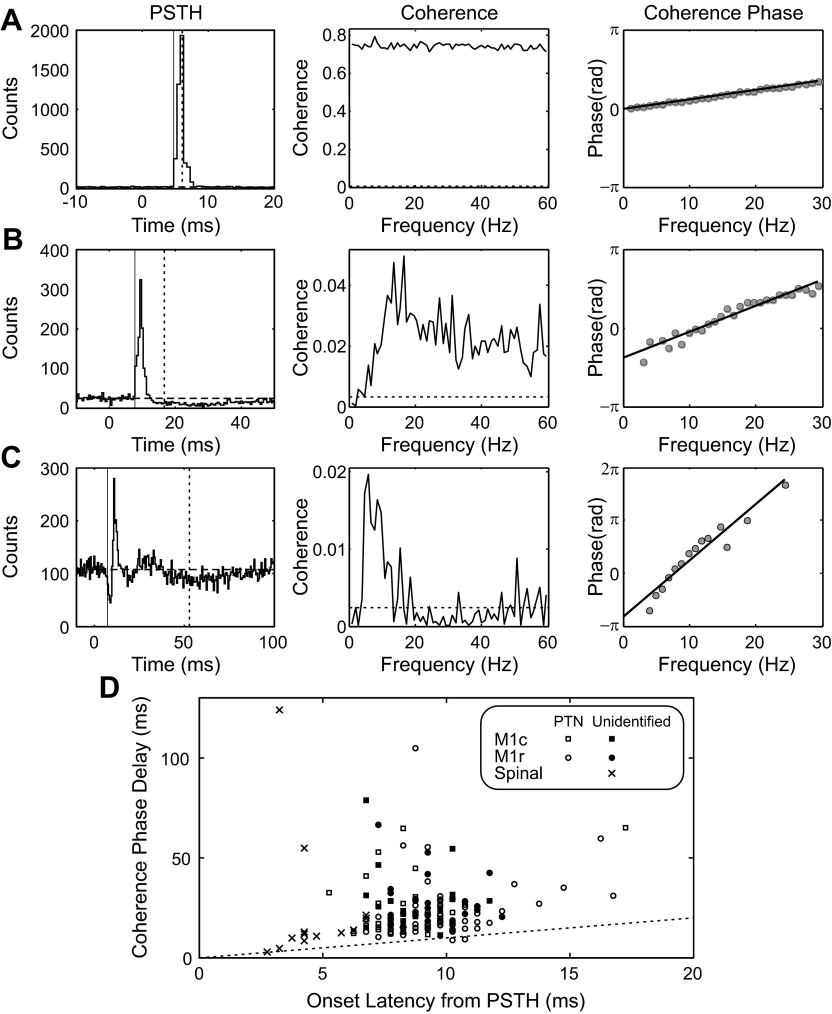
Comparison of delay measurements made from PSTH and coherence phase delay. *A–C*: 3 different neural responses. *Left*: PSTH; thin solid vertical line indicates measured onset latency. *Center*: coherence spectrum; horizontal dashed line indicates significance limit (*P* < 0.05). *Right*: coherence phase spectrum with superimposed linear regression fit. Delay implied by this linear phase-frequency relationship is shown as a vertical dashed line on the PSTH. *A*: spinal cord cell. *B*: unidentified cell from M1c. *C*: PTN from M1c, antidromic latency 4.3 ms. *D*: scatterplot of delays estimated from coherence phase delay vs. PSTH onset latency. Different symbols mark PTNs or unidentified cells, recorded from M1c, M1r, or spinal cord. Dashed line is the line of equality.

When neural responses to a stimulus are characterized in the time domain with a PSTH, it is straightforward to measure the response onset latency. When coherence between a spike train and stimulus is calculated in the frequency domain, it is also possible to compute the coherence phase. For a system that incorporates a fixed delay, the phase-frequency relationship should be linear, with a slope equal to the time delay multiplied by 2π (*Eq. 7*). Figure 7 presents a comparison of these two methods of estimating response timing.

Figure 7*A* illustrates a cell recorded in the spinal cord responding to superficial radial nerve stimulation. This neuron showed a powerful brief facilitation of its spiking following the stimulus, with an onset latency of 4.75 ms (marked by thin vertical line on PSTH of Fig. 7*A*). The coherence was larger than 0.7 for a wide range of frequencies. The dependence of coherence phase on frequency was well fitted by a straight line (*r*^2^ = 0.997), with a slope indicating a delay of 6.1 ms (calculated as in *Eq. 7*). This delay is marked on the PSTH in Fig. 7*A* with a dashed vertical line; it lay closer to the middle of the PSTH peak, rather than its onset.

Figure 7*B* shows the response of an unidentified cell recorded in M1c to median nerve stimulation. This cell exhibited a brief facilitation of its discharge, with onset latency of 4.75 ms; this was followed by a longer-lasting suppression. The coherence was consistently above significance for frequencies higher than 5 Hz. The coherence phase was also closely approximated by a linear relationship with frequency (*r*^2^ = 0.945); the slope implied a delay of 16.6 ms, which lay after the PSTH peak (dashed vertical line). In this case, the delay estimated by the coherence phase-frequency relationship appeared to be sensitive not only to the discharge facilitation but also to the suppression. Although this had low amplitude relative to baseline in the PSTH, its long duration meant that it exerted a considerable influence over the delay estimate determined from the coherence phase.

Figure 7*C* illustrates the response of an identified PTN recorded in M1c to median nerve stimulation. The PSTH in this case revealed multiple response components, beginning with a brief suppression with an onset latency of 7.25 ms. Coherence rose above significance consistently only for frequencies below 20 Hz. The phase-frequency relationship was also well approximated by a straight line (*r*^2^ = 0.923), which yielded a delay estimate of 52.9 ms. Once again, this was influenced by the long-lasting discharge suppression that was the final component of the response.

Figure 7*D* presents a comparison across the recorded population of response onset latencies measured from the PSTH and the delay estimated from the coherence phase-frequency relationship. Results from all stimulated nerves have been pooled for this analysis. As in Fig. 7, *A–C*, coherence phase-frequency regressions were calculated for frequencies up to 30 Hz, using only spectra with at least three phase estimates available over this range (corresponding to 3 frequency bins where coherence was significantly different from 0). In 206 of 258 available spectra (80%), the linear fit to the phase-frequency relationship had a slope significantly different from 0 (*P* < 0.05). The mean *r*^2^ value for these linear fits was 0.85. A total of 197 of 206 spectra (= 96%) had *r*^2^ > 0.6, and 155/206 (= 75%) had *r*^2^ > 0.8, suggesting that most phase-frequency spectra were well fitted by a straight line. Each point in Fig. 7*D* marks a combination of neural spike train and stimulus for which both the response in the PSTH, and also the linear regression of phase versus frequency, were significant. Different symbols mark identified PTNs and unidentified cells recorded from the different M1 subregions and spinal cord. The dashed line indicates the expected relationship if these two measurements were equal. Almost all points lay above the line, showing that the coherence phase delay estimate was usually larger than the PSTH response onset latency. Coherence phase delay estimates thus incorporate all components of the response, rather than being sensitive to just the earliest (onset) latency.

### Phase Differences Between Motor Cortex and Spinal Cord Neurons

**Fig. 8.**
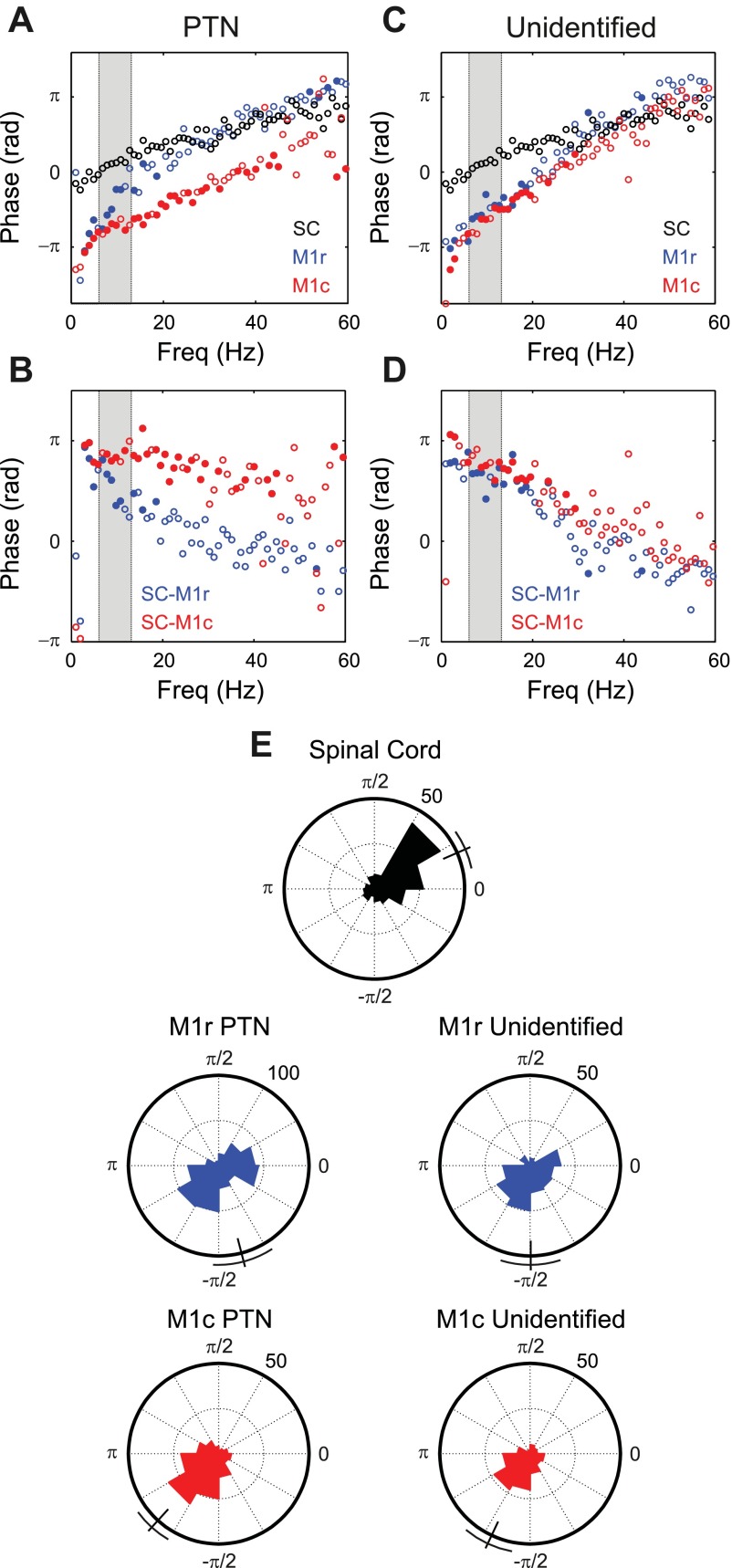
Comparison of coherence phase measurements for spinal and M1 cells responding to ulnar and median nerve stimulation. *A*: circular average coherence phase, over the population of cells with significant coherence at the given frequency. Black, spinal cells; blue, M1r PTNs; red, M1c PTNs. *B*: difference between M1 and spinal cord average phase. Filled symbols in *A* and *B* indicate frequencies at which spinal and M1 phase differ significantly (*P* < 0.05). *C* and *D*: as *A* and *B* but for unidentified cells from each M1 subdivision. *E*: circular histograms indicating the distribution of coherence phase for each region and cell class, over the 6–13 Hz range of physiological tremor indicated by gray shading in *A–D*. Each cell has contributed 1 count to the relevant histogram for every frequency bin with significant coherence in the 6–13 Hz range, for response to median and ulnar nerves. Circular mean phase and its 95% confidence limit are shown as a line and bracket outside each histogram.

An important aim of the present study was to determine whether different phases of response to peripheral inputs in the motor cortex and spinal cord could be responsible for the different phases of firing seen in these centers relative to natural peripheral oscillations during tremor. Accordingly, Fig. 8 presents data on the coherence phase. Results have been combined between the median and ulnar nerves, because both of these nerves supply forearm flexor muscles and receive cutaneous afferents from the palmar side of the hand and coherence and PSTH results from these two nerves appeared similar (Fig. 5, *A* and *B* and Fig. 6, *A* and *B*).

Figure 8*A* shows coherence phase spectra, averaged over spinal cord cells (black) and PTNs from M1r (blue) and M1c (red). Filled symbols indicate frequency bins where the M1 PTNs corresponding to that color had a phase significantly different from the phase of the spinal cord cells (*P* < 0.05, shuffle test as described in methods). Figure 8*B* shows the phase difference between the responses of M1c or M1r PTNs and responses of spinal cord neurons. The response of M1r PTNs to peripheral stimuli differed in phase from that of spinal cord cells over a frequency range from 2.9 to 18.6 Hz (11/17 bins significantly different). The phase difference declined progressively as frequency increased, from 2.94 radians at 2.9 Hz to 1.24 radians at 18.6 Hz; at higher frequencies the M1r PTN and spinal cord response phases seemed to overlie well. By contrast, M1c PTN responses showed significant phase differences from the spinal cord cells over a wide frequency range (29/44 bins significantly different over 2.9–44.9 Hz).

Figure 8, *C* and *D*, show similar plots for M1 unidentified cells. There were significant phase differences between both M1r and M1c populations and the spinal cord, at frequencies from 2.0 to 19.5 Hz. The size of this difference was comparable to that seen for M1c PTNs.

The plots of Fig. 8, *A–D*, represent the frequency relationship of phase well; however, because they average across cells they fail to illustrate whether there is any heterogeneity within the recorded population. As an alternative display, Fig. 8*E* shows the response phases in the 6–13 Hz band for the different regions as circular histograms. In these histograms, each cell contributed one count for every frequency bin where coherence was significant. For both spinal cord cells and M1c PTNs, phases appeared tightly clustered. The mean phases were 0.41 ± 0.21 and −2.31 ± 0.21 radians (circular mean ± 95% confidence limits, shown on histograms as lines and brackets); the difference in mean phase was 2.72 ± 0.29 radians. This was close to, but significantly smaller than, π radians, which would indicate an antiphase relationship. By contrast, for M1r PTNs the response phases appeared bimodally distributed, with peaks approximately corresponding to those seen in M1c and spinal cord. The circular mean phase was −1.31 ± 0.31 radians, a difference of 1.72 ± 0.37 radians relative to the spinal cord responses. Unidentified cells showed relationships similar to the PTNs: M1c, circular mean phase −2.01 ± 0.27 radians (difference from spinal cord 2.42 ± 0.34 radians); M1r, circular mean phase −1.57 ± 0.30 radians (difference from spinal cord 1.98 ± 0.37 radians). As noted above, Fig. 8 combines results from median and ulnar nerves. Sixty-four percent of the phase values illustrated from Fig. 8*E* came from the median nerve; the proportions were similar across the different cell classes (range 58–68%). Similar results were seen when analysis was restricted to data from median or ulnar nerves alone.

The median and ulnar nerves contain a mixture of muscle and cutaneous afferents; it is of interest to determine whether similar phase differences can be seen in the responses to the purely cutaneous superficial radial nerve. Unfortunately, for M1c PTNs (which showed the clearest phase differences relative to the cord for median and ulnar nerves), coherence values for responses to the superficial radial nerve were very low (Fig. 6*D*). This led to few estimates of phase, and considerably more variability in the estimates available for this cell class. We should therefore be cautious in placing too much weight on these results. However, despite this we did find significant phase differences between the response to superficial radial nerve stimuli of spinal cord cells (circular mean phase 0.18 ± 0.4 radians) and both M1c PTNs (circular mean phase −1.44 ± 1.58 radians) and M1c unidentified cells (circular mean phase 1.77 ± 1.57 radians). There were no significant differences between coherence phases for spinal cord neurons and either M1r PTNs (circular mean phase 0.01 ± 0.64 radians) or unidentified cells (circular mean phase 1.00 ± 1.57 radians).

The results from this section indicate that, over the 6–13 Hz range, many cells within M1 fire at approximately the opposite phase relative to peripheral input compared with cells in the spinal cord. Convergence of sensory responses from M1 and the spinal cord onto motoneurons would thus lead to partial phase cancellation, and attenuation of signal amplitude in the frequency range important for physiological tremor.

### Response of Spinal Cells to Pyramidal Tract Stimulation

**Fig. 9.**
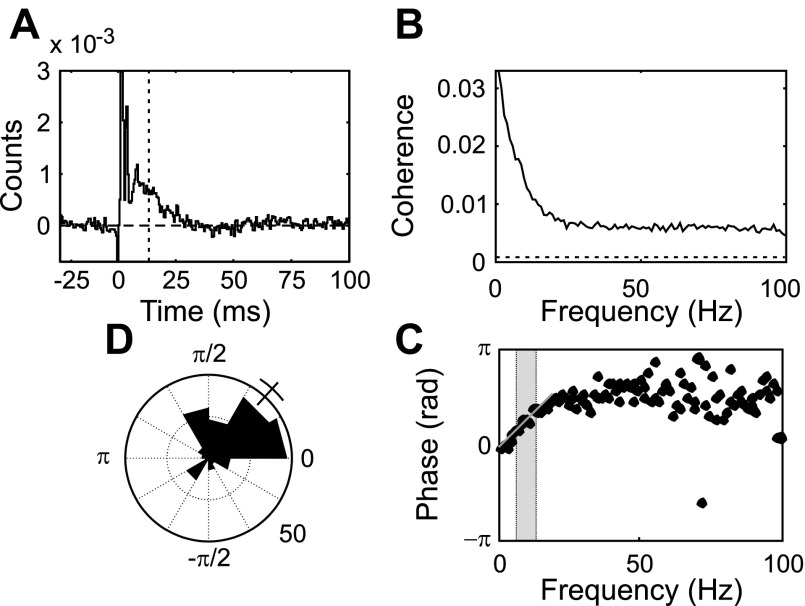
Response of spinal cord neurons to pyramidal tract stimulation. *A*: averaged PSTH, normalized as described in Fig. 5. *B*: averaged coherence spectrum. Dashed line indicates significance limit on averaged coherence (*P* < 0.05). *C*: circular average of coherence phase. The average for each frequency bin includes only cells with significant coherence at that frequency. Gray shading indicates 6–13 Hz range of physiological tremor. Gray line indicates linear regression fit to phase values below 20 Hz. The delay estimated from the slope of this line is marked as a vertical dashed line in *A*. *D*: circular histogram of individual coherence phase values. Each cell has contributed 1 count for every frequency bin with significant coherence in the 6–13 Hz range. Circular mean phase and its 95% confidence limit are shown as a line and bracket outside the histogram.

Many spinal cord interneurons receive input from the corticospinal tract. One way in which cortical and spinal activity could exhibit an antiphase relationship is if spinal cells responded to corticospinal input with a profile that introduced the necessary phase shift. We investigated this by recording spinal responses to PT stimulation, with stimuli timed as a Poisson train as above. Figure 9*A* illustrates the average PSTH of spinal cells after such stimulation. There was a powerful facilitation that had an onset latency of only 0.75 ms. Figure 9*B* illustrates the corresponding average coherence spectrum; coherence declined up to 20 Hz but remained above significance for all frequencies illustrated. The average phase spectrum exhibited a significant linear phase-frequency relationship below 20 Hz, with a slope corresponding to a delay of 13.4 ms (gray line, Fig. 9*C*; this delay is marked as a vertical dashed line in Fig. 9*A*). At higher frequencies, the phase appeared approximately constant (mean for 30–100 Hz range of 1.67 radians). Figure 9*D* illustrates the distribution of phase in the 6–13 Hz range. The mean phase was 0.83 ± 0.16 radians (circular mean ± 95% confidence limit). These results indicate that only small phase shifts are introduced by the connections between corticospinal tract and spinal interneurons.

## DISCUSSION

In this report, we have shown important differences between M1 and spinal cord neurons in the phase relationship of neural spiking to peripheral input. Below, we consider the possible underlying reasons for these differences, as well as their implications for motor control.

### Motor Cortical Responses to Peripheral Inputs

A considerable body of previous work has described responses to sensory input in M1 cells. This previous work focused on two main issues. The first was the pathway over which sensory information might reach the motor cortex. The results of focal lesions suggest that the dorsal columns are the main ascending tract carrying peripheral inputs to M1 (Asanuma et al. 1980). Lesions of the primary somatosensory cortex (S1) reduce, but do not completely abolish, M1 sensory responses (Asanuma et al. 1980). M1 receives input both from S1 (Jones et al. 1978) and also directly from the thalamus (Lemon and van der Burg 1979).

A second important strand of previous work has been to characterize the submodality of inputs to M1. Several studies suggest that different parts of M1 receive submodality-specific input. One representation of the body within the bank of the central sulcus (M1c) is reported to receive almost exclusively cutaneous input, whereas the more superficial part (M1r) has a predominance of deep responses, probably from muscle or joint afferents. These results have been described in both anesthetized New World monkeys (Strick and Preston 1982) and awake macaques (Tanji and Wise 1981). In the present data, we found coherence between cells in M1r and stimulation of the purely cutaneous superficial radial nerve, whereas for M1c PTNs the coherence was at chance levels (Fig. 6*D*). This is the opposite of what is expected in light of the previous work.

However, many other reports yield a more mixed picture of input distribution. Rosen and Asanuma (1972) and Wong et al. (1978) recorded cells with cutaneous inputs in both surface and deep cortical locations. Lemon and Porter (1976) found that cutaneous inputs were confined to the bank of the sulcus, whereas neurons with deep receptive fields were widespread. Cheney and Fetz (1984) reported that corticomotoneuronal cells identified by spike-triggered averaging were strongly activated by perturbations of the wrist joint induced by a torque pulse. Such a stimulus would produce activation of many receptor classes; however, for the small number of cells that could be tested in detail no cutaneous receptive field could be located, suggesting that a major component of these responses was mediated by muscle receptors. Although no data on the anatomical location of these cells were provided, recent work suggests that the majority of corticomotoneuronal cells are located in the bank of the sulcus (M1c; Rathelot and Strick 2006, 2009).

It thus appears that there is no absolute division of the submodality of somatosensory input received by M1c versus M1r. Any differences between findings are likely to reflect the difficult and subjective task of categorizing responses. Tanji and Wise (1981) and Lemon and Porter (1976) both noted the difficulty of detecting additional joint input to a cell that had a cutaneous receptive field. Wiesendanger (1973) took the different approach of using electrical nerve stimulation—as here—and also reported that many M1 cells responded to both deep and superficial radial nerve stimulation. The differences between the average coherence of M1c and M1r neurons with superficial radial nerve stimulation in our study probably reflect fluctuations in individual inputs among the relatively small number of neurons sampled rather than a consistent finding. In addition, the coherence analysis used here is sensitive to all features of responses, including discharge suppression, whereas previous studies that located receptive fields by clinical testing necessarily emphasized stimulus-locked increases in rate.

### Timing of Responses to Peripheral Input

Our previous work reported that M1 PTNs and spinal cord interneurons fired in antiphase relative to voluntary finger movements in the 6–13 Hz range of physiological tremor (Williams et al. 2010), which could lead to cancellation of oscillatory input at motoneurons. This would have the consequence of reducing tremor, with subsequent improvements in motor performance. However, that study was conducted in awake animals producing a voluntary movement, in which all feedforward and feedback neural control systems were intact. It was thus impossible to determine unambiguously what neural circuitry might be responsible for the antiphase firing of spinal cord relative to motor cortex. One possibility is that spinal circuits simply phase invert oscillatory descending commands from the cortex. Another possibility is that spinal and cortical circuits respond differently to a source of oscillations from another center.

In the present work, we have partially opened the feedback loop by delivering stimuli with timing not dictated by voluntary motor output. Spinal cord interneurons responded to electrical stimulation of the PT with a mean phase in the 6–13 Hz range of only 0.26π radians (Fig. 9*D*), suggesting that spinal circuits are not capable of phase-inverting corticospinal input. By contrast, inputs from peripheral nerve activated M1c PTNs and spinal cord interneurons with phase relationships that differed by 0.87π radians over a wide range of physiologically relevant frequencies (Fig. 8). Antiphase firing between M1 and the spinal cord during voluntary movements is thus likely to result in large part from different responses to oscillations in afferent input. Supporting a dominant role for afferent feedback, we previously showed with directed coherence analysis that M1 oscillations during tremor were mainly driven by peripheral oscillations, rather than driving them (Williams et al. 2009).

Although phase differences were clearest between spinal cord interneurons and M1c PTNs, significant differences were also seen for M1r PTNs. On average, while the phase difference for M1c relative to the spinal interneurons was slightly lower than π, that for M1r was around π/2. However, the average phase poorly reflected the underlying population, which seemed bimodally distributed (Fig. 8*E*). Around half of the M1r PTNs showed a phase relationship with sensory input similar to the spinal cord, while the other half fired close to antiphase with the cord, like M1c PTNs. Recent anatomical work has suggested that M1c contains the majority of corticomotoneuronal cells, which provide direct input to motoneurons (Rathelot and Strick 2006, 2009). By contrast, corticospinal output from M1r seems to act largely via connections to spinal cord interneurons. The phase relationships of M1r with sensory input may thus reflect in part responses to strong cortico-cortical input from M1c and in part a local transformation of this input to bring responses closer to those of the spinal interneurons that are the target of M1r output. Unidentified cells in both M1c and M1r showed a relationship to peripheral input compared with the spinal cord that was intermediate between π and π/2 (0.77π and 0.62π radians difference in circular mean phases, respectively; Fig. 8, *D* and *E*).

Several methodological aspects of the present study should be considered, as they may have influenced our results. First, the animals were anesthetized or sedated during recordings. While this was essential to exclude contributions from voluntary movements, it is possible that the response profiles that we measured were different from those that would occur in the awake state. Anesthetics can increase the levels of spontaneous oscillatory behavior. This might lead to resonance phenomena between the stimulated nerve input and cell activity (Williams and Baker 2009a), which can produce some changes in response phase. We sampled only cells that were spontaneously active under anesthesia; we cannot determine how representative these are of the active populations in the awake state. The anesthetic regime differed for the spinal and cortical recordings, which could have resulted in some of the response differences observed. In addition, we used electrical stimulation of peripheral nerves; this generates a highly synchronous afferent volley, unlike the more asynchronous sensory input that would occur during normal behavior. Conversely, we activated the four peripheral nerves with independent Poisson train stimuli; during natural behavior, activity in these nerves would be more or less correlated according to the task at hand. Electrical stimuli were just below motor threshold, which would activate only large-fiber group I afferents. Contributions from slower-conducting afferents are also likely to be important to the overall sensory responses, although as these show more dispersion in their conduction velocities, they might be less effective at encoding peripheral oscillations at tremor frequencies. We cannot exclude an important influence of any of these factors on our results. However, it is striking that we find a close to antiphase difference in sensory responses of the cortical and spinal cord neurons, which is only slightly smaller than the phase difference seen in their spontaneous firing during natural behavior. This suggests that sensory feedback is likely to play a dominant role in generating the antiphase activity in the awake state, in agreement with our previous results from directed coherence analysis (Williams et al. 2009).

If antiphase spinal and cortical activity are to lead to phase cancellation and potential reduction of output at tremor frequencies, both spinal and cortical neurons must be excitatory to motoneurons. We have demonstrated in our work with awake behaving animals that at least some of the spinal cord interneurons recorded were excitatory (Williams et al. 2010), and hence the antiphase spinal activity recorded there should produce phase cancellation. Such identification rests on spike-triggered averaging of the electromyogram; this was not possible in our sedated animals, in which the muscles were relaxed. However, as recordings were made with the same approach and electrode types as previously, it seems unlikely that a very different population was sampled in the present work.

An important question concerns the mechanisms underlying the differences in neural response phase. Previous work has characterized response timing with onset latency and has used this to estimate feedback loop delays and the associated phase at a given frequency. By this approach, there would seem to be little reason to expect phase differences as large as we observed. Population onset latencies differed by only 2 ms between M1c and spinal cord, which corresponds to only 2% of an oscillation period at 10 Hz. However, the response to an oscillatory input is affected by the entire neural response profile, and not simply its earliest component (Riddle and Baker 2005; Williams and Baker 2009a). Later components of the neural response, including periods of suppression, critically influence the ability of a neuron to encode information at a given frequency (coherence magnitude), as well as the timing of that information transmission (coherence phase). In the somatosensory evoked potential literature, the earliest response is assumed to reflect a direct response to afferent input by primary sensory areas, whereas later components reflect recurrent processing of the input by intracortical circuits including higher cortical areas (Allison et al. 1989). Similar considerations must apply to the unit responses recorded here, suggesting that the different phases of neural response seen in M1 PTNs versus spinal cord interneurons reflect differences in neural circuits providing recurrent excitation and inhibition. Since such late response components reflect a network of multiple synaptic connections, they are more likely to be amenable to change following plasticity in intracortical or intraspinal connections than the initial part of the response, which is mediated by more direct (oligosynaptic) routes. This raises the interesting possibility that they could be continually adjusted to optimize phase for maximal cancellation of unwanted oscillations. Plasticity of synapses has previously been demonstrated to play a key role in the synchronization behavior of coupled networks (Bibbig et al. 2002).

Convergence of two antiphase signals may lead to cancellation; however, if the system is nonlinear or the signals nonsinusoidal, period doubling is another possibility. Although these phenomena are distinct when considering neural activity, the functional consequences for movement may be similar. Whereas oscillations around 10 Hz are within the pass band of the musculoskeletal apparatus, those around 20 Hz are usually above it (Burke 1981; Elble and Koller 1990; Williams and Baker 2009b) and hence have minimal effect on motor output. Period doubling that resulted in a frequency shift of oscillations up to 20 Hz could thus reduce tremor as effectively as phase cancellation.

Early studies on tremor drew a distinction between tremor generated by oscillations in a peripheral feedback loop, such as the monosynaptic stretch reflex arc, and tremor produced by centrally generated oscillations (Elble and Koller 1990). A parallel might be drawn with earlier studies of motor control, where spinal reflex responses to sensory information were contrasted with cortical voluntary motor output. More recent concepts have emphasized the need to integrate sensory feedback into the optimal planning and correction of voluntary movements (Todorov and Jordan 2002). Within this framework, the antiphase firing of spinal cord and M1 cells that we have described can be viewed as permitting effective feedback control of movement while removing the propensity for damaging oscillations that such feedback might produce. Several previous papers have also proposed that responses of the central nervous system might improve the stability of reflex responses. Hore and Flament (1986) provided evidence that the cerebellum reduces oscillations produced by sensory feedback. Matthews (1997) showed that the biophysical properties of motoneurons could introduce a phase advance into the monosynaptic stretch reflex, also leading to improved stability around the frequencies of physiological tremor. Finally, a modeling study from this laboratory showed that recurrent inhibition from Renshaw cells was capable of reducing oscillations in motor output at the frequencies of physiological tremor (Williams and Baker 2009b).

Our results show that ∼10-Hz oscillations detected by peripheral afferents will be partially canceled by superimposition of M1 and antiphase spinal cord responses converging on motoneurons. The extent of this cancellation will depend on how well matched the amplitudes of sensory responses conveyed to motoneurons via M1 and the spinal cord are. In the present work, we showed that coherence between sensory input and cell discharge was of similar amplitude for M1 and spinal neurons (Fig. 6). No quantitative data are available on the relative magnitudes of synaptic input to motoneurons from these two sources, which would also need to be similar to allow effective phase cancellation. We have previously shown that trial-by-trial fluctuation in tremor amplitude can be partially explained by fluctuations in the relative amplitude of spinal and M1 oscillations (Williams et al. 2010), suggesting that amplitudes are sufficiently well matched that some effective cancellation can occur.

As well as oscillatory input from the periphery, spinal cord intrinsic circuits (Lidierth and Wall 1996) and thalamocortical systems (Hughes and Crunelli 2005) both appear capable of generating oscillations around 10 Hz. We have shown that corticospinal input fires spinal cord interneurons with little phase lag (Fig. 9). This might suggest that oscillations originating in the cortex will be passed by spinal cord interneurons with minimal phase shift to motoneurons, allowing summation without phase cancellation. In that case, only tremor of peripheral origin would be reduced by the mechanism that we have investigated. However, the situation is likely to be substantially more complex. As one example, previous modeling work shows that two neural oscillatory centers may be synchronized together if they are reciprocally coupled, either by long-range inhibitory connections or (of more relevance to corticospinal circuits) by long-range excitation that recruits local inhibition (Bibbig et al. 2002; Ernst et al. 1995; Gerstner et al. 1996; Van Vreeswijk et al. 1994). In this case, two stable modes exist: one where the centers fire in phase and another antiphase. Such phase relationships are independent of the neural conduction delays between the two centers. We speculate that differences in peripheral responses between spinal cord and M1 reported here may bias them toward an antiphase oscillatory coupling. In that case, all oscillatory activity close to tremor frequencies would be subject to phase cancellation between spinal and M1 inputs to the motoneurons, and not just that of peripheral origin.

The latter considerations may explain a recent observation by Mendez-Balbuena et al. (2012), who showed that the addition of a low level of noise in somatosensory input could reduce the variability in motor output. We speculate in this case that enhanced sensory input might have switched spinal and M1 circuits from an in-phase to an antiphase coupling, leading to phase cancellation around tremor frequencies and attendant more precise output. Further understanding the interactions between sensory feedback and centrally generated oscillations might open up novel avenues for treatment of pathological tremors, which remain the most common neurological sign.

## GRANTS

This work was funded by the Wellcome Trust.

## DISCLOSURES

No conflicts of interest, financial or otherwise, are declared by the author(s).

## AUTHOR CONTRIBUTIONS

Author contributions: S.K. and S.N.B. performed experiments; S.K. and S.N.B. analyzed data; S.K. and S.N.B. edited and revised manuscript; S.K. and S.N.B. approved final version of manuscript; S.N.B. conception and design of research; S.N.B. interpreted results of experiments; S.N.B. prepared figures; S.N.B. drafted manuscript.
